# Sequencing and characterization of the complete mitochondrial genome of the endangered Devils Hole pupfish *Cyprinodon diabolis* (Cyprinodontiformes: Cyprinodontidae)

**DOI:** 10.1080/23802359.2016.1225526

**Published:** 2016-09-18

**Authors:** Sean C. Lema, Kevin P. Wilson, Brandon L. Senger, Lee H. Simons

**Affiliations:** a Biological Sciences Department, California Polytechnic State University, San Luis Obispo, CA, USA;; b Pahrump Field Office, Death Valley National Park, Pahrump, NV, USA;; c Nevada Department of Wildlife, Las Vegas, NV, USA;; d Southern Nevada Fish and Wildlife Office, U.S. Fish and Wildlife Service, Las Vegas, NV, USA

**Keywords:** Fish, mitochondria DNA, conservation, Endangered species, genetics

## Abstract

The Devils Hole pupfish (*Cyprinodon diabolis*) is an imperiled cypinodontid fish endemic to Devils Hole, a single groundwater-fed pool in western North America characterized by temperature and oxygen conditions lethal to most fishes. In this study, we obtained the 16,499 base pair (bp) mitochondrial DNA sequence of *C. diabolis*. The mitogenome encodes 13 polypeptides, 22 tRNAs, 12S and 18S rRNAs, and an 832 bp D-loop region, and has a nucleotide composition of A, 25.69%; T, 27.32%; G, 17.49%; and C, 29.50% (GC content: 46.99%). The availability of this mitogenome will facilitate evaluations of *C. diabolis* genetic structure for management and conservation of this iconic endangered species.

The Devils Hole pupfish *Cyprinodon diabolis* (Wales 1930) is a small cyprinodontiform fish belonging to a clade of allopatric pupfishes that evolved in the Death Valley region of the Mojave Desert in southern Nevada, USA (Miller 1948, 1950; Duvernell & Turner 1998; Echelle 2008; Martin et al. 2016). *Cyprinodon diabolis* occupies what may be the smallest range of any vertebrate (Moyle 2002): Devils Hole, a single geothermal pool having high temperature (∼33.5 °C) and low dissolved oxygen (ambient 2.5 mg/l) conditions (Soltz & Naiman 1978; Riggs & Deacon 2002) and variable nutrient influx that limits population size (Bernot & Wilson 2012; Hauser et al. 2014). *Cyprinodon diabolis* was listed under the Endangered Species Preservation Act of 1966 (Federal Register 1967) and grandfathered into the United States’ Endangered Species Act. The population declined sharply with local groundwater pumping during the 1970s (Deacon & Williams 1991; Stoike and Pister 2010). Since that time, the species experienced population bottlenecks including a low population size of 35 fish in 2013. Conservation efforts have aimed to recover *C. diabolis* in Devils Hole and establish additional populations in artificial refuges (Sharpe et al. 1973; Baugh & Deacon 1988; Karam et al. 2012), although past efforts were impeded by genetic and phenotypic changes to refuge populations (Williams 1977; Wilcox & Martin 2006; Martin et al. 2012).

Here, we report the complete mitochondrial DNA genome of *C. diabolis* (Genbank accession no. KX061747) from a deceased individual recovered on 5 November 2015 (26 mm total length, collected at 21:00 h by B. Senger) from Devils Hole, Nevada, USA (36°25′31″N, 116°17′29″W). DNA was isolated from skeletal muscle (DNeasy Blood and Tissue Kit, Qiagen, Valencia, CA), and overlapping regions of the mtDNA were amplified using GoTaq^®^ Long PCR Master Mix (Promega Corp., Madison, WI) and primers designed previously for sequencing the mitogenome of *C. variegatus* (Barcelon & Lema 2016). The resulting PCR products were Sanger sequenced (MC Lab, South San Francisco, CA) and assembled (Sequencher v5, Gene Codes Corp., Ann Arbor, MI).

The mitochondrial genome of *C. diabolis* is 16,499 bps and encodes 13 protein subunits in the arrangement typical of Actinopterygiian mitogenomes, with all protein-coding genes located on the heavy strand (H-strand) except NADH dehydrogenase subunit-6 (*nd6*). The mitogenome also includes 22 tRNAs and two rRNAs (12S and 18S) with the following non-coding RNAs on the light strand (L-strand): *tRNA^Gln^*, *tRNA^Ala^*, *tRNA^Asn^*, *tRNA^Cys^*, *tRNA^Tyr^*, *tRNA^Ser^*, *tRNA^Glu^*, and *tRNA^Pro^*. Phylogenetic analysis confirmed close evolutionary relationships of *C. diabolis* to two other pupfishes from the Death Valley region: *C. nevadensis pectoralis* (KP064222; Keepers et al. 2016) and *C. n. amargosae* (KU883631) (Figure 1). Despite the morphological and behavioural distinctiveness of *C. diabolis* (Miller 1948, Soltz & Naiman 1978), the *C. diabolis* mitogenome exhibited 99.436% and 99.382% nucleotide identity, respectively, to the *pectoralis* and *amargosae* subspecies of *C. nevadensis*. Even so, missense mutations that changed amino acid properties are present between *diabolis* and *nevadensis* in genes encoding cytochrome c oxidase subunit 2 (*cox2*) and NADH dehydrogenase subunits *nd1*, *nd2*, *nd3*, and *nd4*.

**Figure 1.**
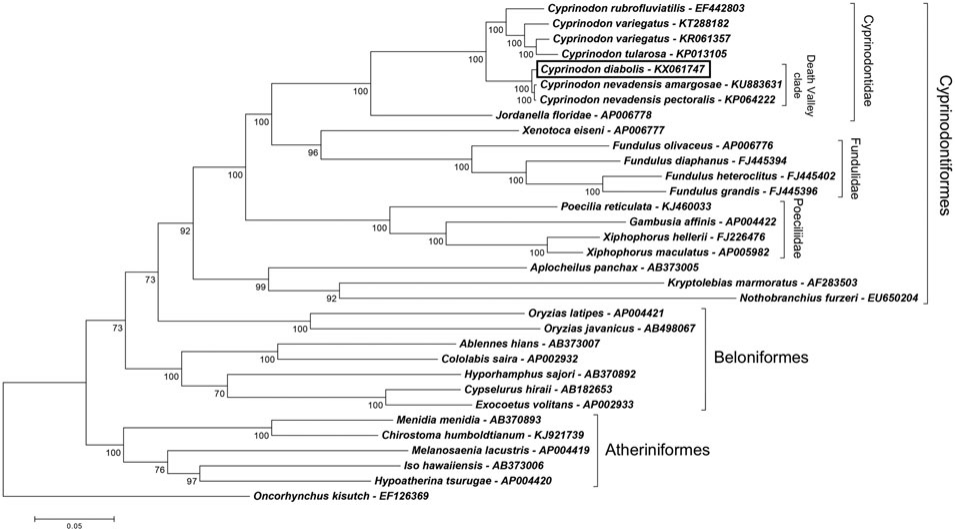
Phylogenomic relationships of *C. diabolis* (KX061747) and other Cyprinodontiformes fishes, as well as select taxa from the orders Beloniformes and Atheriniformes. Nucleotide sequences were aligned with Clustal X software (Larkin et al. 2007), and the maximum-likelihood tree was assembled using a Tamura-Nei Model with all sites (MEGA v7 software; Kumar et al. 2016). Mitogenomes were completed for all taxa except *Jordanella floridae*, which lacked a D-loop region. Bootstrap values (1000 replicates) are indicated at each node. The coho salmon (*Oncorhynchus kisutch*, order Salmoniformes) was used as an outgroup. Pupfishes from the Death Valley geographic region (Death Valley clade) are indicated within the family Cyprinodontidae. GenBank accession numbers accompany each taxon. Note that order Beloniformes is not monophyletic in the phylogeny.
